# Determination of iris thickness development in children using swept-source anterior-segment optical coherence tomography

**DOI:** 10.1371/journal.pone.0217656

**Published:** 2019-05-28

**Authors:** Shunsuke Nakakura, Yuki Nagata, Yukiko Shimizu, Akiko Kawai, Hitoshi Tabuchi, Yoshiaki Kiuchi

**Affiliations:** 1 Department of Ophthalmology, Saneikai Tsukazaki Hospital, Himeji, Japan; 2 Department of Ophthalmology and Visual Sciences, Graduate School of Biomedical Sciences, Hiroshima University, Hiroshima, Japan; Massachusetts Eye & Ear Infirmary, Harvard Medical School, UNITED STATES

## Abstract

**Purpose:**

The uvea comprises the iris, ciliary body, and choroid. However, the development of the anterior part (iris and ciliary body) in children is not yet fully elucidated. We investigated the iris thickness (IT) in children using swept-source anterior-segment optical coherence tomography (ASOCT).

**Methods:**

In this retrospective, clinic-based study, we enrolled 41 children (mean ± standard deviation: 6.8 ± 3.3 years; range: 3–16; 17 males) with normal or mild refractive error. Horizontal scanning images of swept-source ASOCT were analyzed in temporal and nasal angle areas. The ITs at 1 and 2 mm from the pupil edge were measured using swept-source ASOCT. The association between IT and age, sex, and ocular morphological parameters (i.e., axial length, average corneal curvature, central corneal thickness, inter-scleral spur distance, and anterior chamber depth) was assessed using Pearson’s correlation coefficient (r) and linear regression analysis.

**Results:**

The average IT (temporal and nasal) at 1 and 2 mm were 0.432 ± 0.060 (0.302−0.569 mm) and 0.337 ± 0.045 (0.229−0.414 mm), respectively. There was a significant correlation between age and average IT (*r* = 0.45, P = 0.002 at 1 mm and *r* = 0.31, P = 0.042 at 2 mm). Multiple linear regression analysis revealed that age (coefficient: 0.01), axial length (−0.02), average corneal curvature (0.01), and anterior chamber depth (0.01) at 1 mm as well as age (0.00), average corneal curvature (0.09), anterior chamber depth (0.06), and male (–0.02) at 2 mm were identified as predictors of IT.

**Conclusions:**

IT in children increases with age. Additionally, IT was thinner with longer axial length and in males, thicker in eyes with deeper anterior chamber and flatter corneal curvature. Our study may partly explain the development of eyeball structures in children.

## Introduction

The uvea consists of the iris, ciliary body, and choroid. Precedingly, the choroidal thickness (CT) was investigated through the development of swept-source optical coherence tomography, leading to new choroidal pathophysiology, such as pachychoroid disease.[1–3] The structural variation/changes and developmental processes of the anterior component of the uvea (i.e., the iris and ciliary body) have not well investigated. The ciliary body is directly invisible and can be precisely evaluated through ultrasound biomicroscopy. However, the iris is directly visible through slit-lamp examination, and noninvasively and rapidly evaluated using anterior-segment optical coherence tomography (ASOCT). Especially, iris thickness (IT) (i.e., CT) is a simple morphological parameter to determine the pathology of the iris. A previous study showed that the IT decreased in patients with Fuch’s uveitis,[4] pseudoexfoliation syndrome,[5] neovascular glaucoma,[6,7] and primary congenital glaucoma.[8] In contrast, it increased in patients with sympathetic ophthalmia.[9] In healthy older patients, the IT varied among different iris colors, but not among different ages.[10,11] Sng CC et al. reported that the IT was not associated with other ocular and demographic parameters in older patients.[11] However Jin P et al. first reported an increase in IT in parallel with the increasing age of children (7–15 years).[12] The association between IT changes and age or other ocular parameters has not been investigated. Therefore, our study aimed to investigate the IT in children and determine the association between IT and ocular parameters, especially in those children who were also in developing period, thus, this study may lead to an understanding of ocular structural development.

## Materials and methods

**Fig 1:**
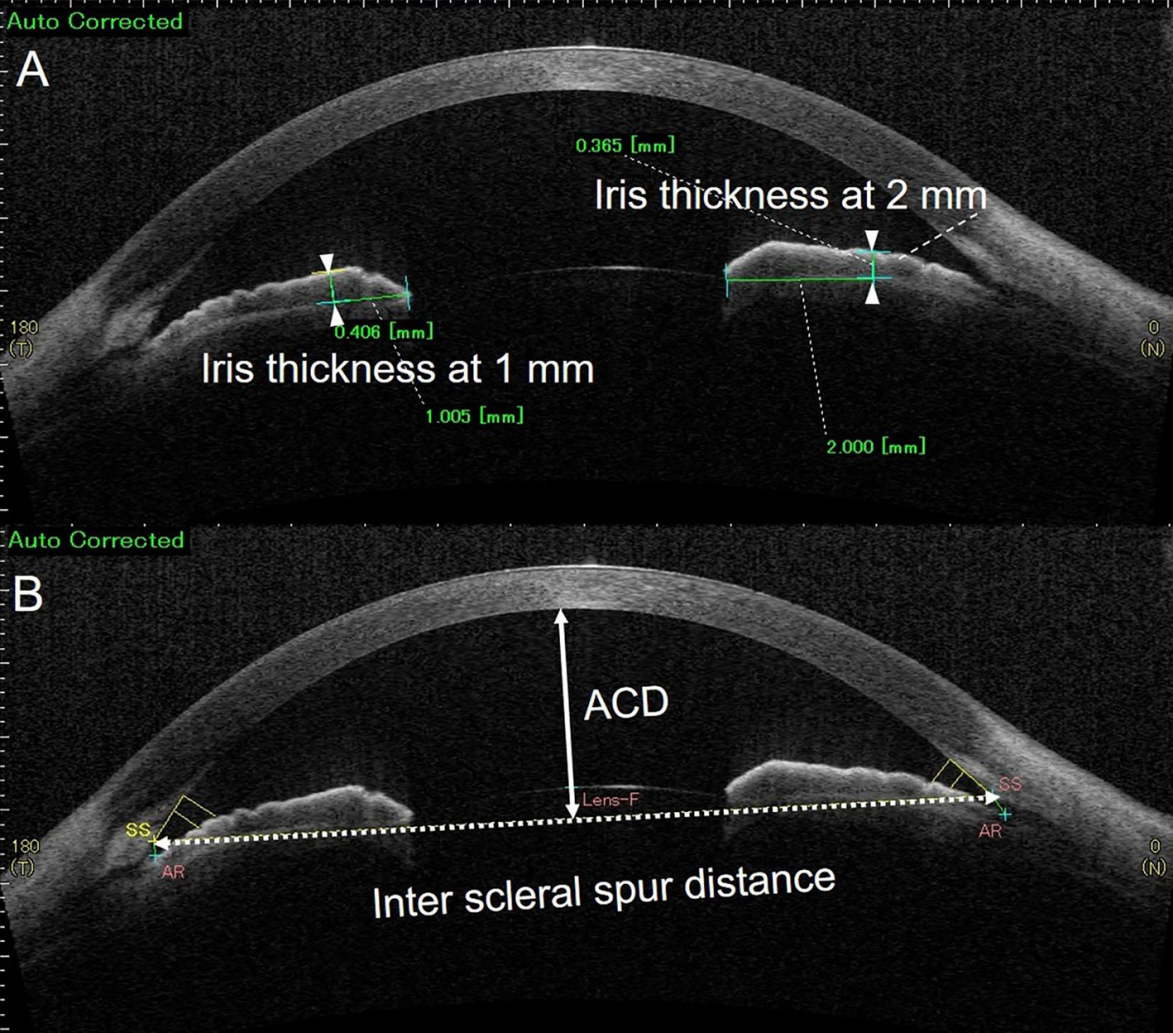
Morphological parameters of the iris and anterior chamber measured using swept-source anterior-segment optical coherence tomography. A. IT (iris thickness) measured at 1 and 2 mm from both the temporal and nasal angles. B. ACD (anterior chamber depth) refers to the vertical width of the anterior chamber. Inter-scleral spur distance (SS distance) refers to the horizontal width of the anterior chamber.

This study was approved by the Institutional Review Board of Saneikai Tsukazaki Hospital (assignment No: 181045) and performed according to the tenets of the Declaration of Helsinki. The IRB granted a waiver of informed consent for this study based on the ethical guidelines for medical and health research involving human subjects established by the Japanese Ministry of Education, Culture, Sports, Science, and Technology and by the Ministry of health, Labor, and Welfare. The waiver was granted because the study was a retrospective chart review, and not an interventional study, as well as because it was difficult to obtain consent from patients who had been treated several years previously. Personal identifiers were removed from the records prior to data analysis. We retrospectively reanalyzed the ASOCT data obtained from children, previously reported previously by Y. Shimizu et al.[13] The patients were children treated at our hospital between October 2012 and July 2013, and without any history of prematurity, ocular surgery. To evaluate the healthy children, we excluded the patients with strabismus and amblyopia. Only the right eyes were analyzed, and the used ocular morphological parameters were as follows. The corneal radius of the curvature (i.e., corneal curvature) was measured using an Auto Kerato-Refractometer (KR-8900; TOPCON, Tokyo, Japan). Ten measurements were used to determine the average corneal curvature (mean of K1 and K2) (mm). The axial length was measured using an IOLMaster 700 (Carl Zeiss Meditec, Jena, Germany), and the mean of five measurements was used for subsequent analyses. We reanalyzed the swept-source ASOCT data (SS-1000 CASIA^TM^, TOMEY, Nagoya, Japan) obtained from 43 children that were taken in the dark room condition without pupil dilation.[12] Vertical scan images in ASOCT were often disturbed by the eyelids; thus, we selected the clearest horizontal scan images. The ASOCT parameters were evaluated by Y.N. in high-resolution two-dimensional mode using software calipers in the SS-1000. In our analysis, the IT was measured from the edge of the pupil in line with the hyperreflective line at the bottom of the iris. The IT was measured twice at 1 and 2 mm (± 0.005 mm) from the edge of the pupil (Fig 1A) used in our previous study,[6,7] and the mean of two measurements was used for subsequent analysis. The eyes of children with large mydriasis in the dark room condition were not suitable for measuring IT because the distal point (2 mm) from the edge of the pupil will locate the angle or cornea. Therefore, two children were excluded from the analysis.

Subsequently, the central corneal thickness (CCT) (μm) and anterior chamber depth (ACD) (mm) were automatically calculated using the built-in software program of the SS-1000. The inter-scleral spur (SS) distance was calculated using software calipers (Fig 1B). The ACD was defined as the distance from the corneal endothelium to the anterior lens.

### Statistical analysis

Statistical analyses were performed using the JMP software version 10.0.0 (SAS Institute Inc., Cary, NC, USA) and the statistical programming language R (version 3.1.3; R Foundation for Statistical Computing, Vienna, Austria). The association between IT and parameters was evaluated using Pearson’s correlation coefficient (*r*). The association between IT and age, sex, and ocular morphological parameters (i.e., axial length, average corneal curvature, CCT, SS distance, and ACD) was assessed using linear regression analysis based on the corrected Akaike Information Criteria index. Subsequently, model selection using the second-order bias-corrected Akaike information criterion (AICc) index from all 2^7^ patterns consisting of the seven variables was performed to identify the optimal regression model. In a regression model, the degrees of freedom decrease as the number of variables increase. Hence, model selection methods are useful when the number of variables is large.[14,15] The AICc provides an accurate estimation even in analyses with a small sample size. [16] The data are expressed as mean ± standard deviation (range). P < 0.05 denoted statistical significance.

## Results

We eventually analyzed 41 children aged 3–16 years (mean ± standard deviation: 6.8 ± 3.3 years). The characteristics of patients are shown in Table 1. The majority were male (17 patients; 41%), mean axial length was 22.3 ± 0.8 mm, mean CCT was 558.4 ± 32.8 μm, mean ACD was 3.0 ± 0.2 mm, and mean SS distance was 11.6 ± 0.3 mm (all data were available as S1 File).

**Table 1:** Patient characteristics.

	All (N = 41)
Age (y)	6.8 ± 3.3 (3–16)
Sex (male, %)	17 (41)
Axial length (mm)	22.3 ± 0.8 (20.6–23.9)
Average corneal curvature (mm)	7.7 ± 0.2 (7.2–8.1)
CCT μm)	558.4 ± 32.8 (503–635)
ACD (mm)	3.0 ± 0.2 (2.4–3.7)
SS distance (mm)	11.6 ± 0.3 (10.9–12.4)
Iris thickness (mm)	
Temporal angle	
Iris thickness at 1 mm (mm)	0.405 ± 0.071 (0.226–0.540)
Iris thickness at 2 mm (mm)	0.336 ± 0.063 (0.206–0.459)
Nasal angle	
Iris thickness at 1 mm (mm)	0.459 ± 0.067 (0.266–0.617)
Iris thickness at 2 mm (mm)	0.338 ± 0.051 (0.224–0.447)
Average of temporal and nasal angle	
Iris thickness at 1 mm (mm)	0.432 ± 0.060 (0.302–0.569)
Iris thickness at 2 mm (mm)	0.337 ± 0.045 (0.229–0.414)

CCT, central corneal thickness; ACD, anterior chamber depth; SS distance, inter-scleral spur distance

The IT values measured using ASOCT are also shown in Table 1. In the temporal angle area, at 1 and 2 mm, the IT values were 0.405 ± 0.071 and 0.336 ± 0.063 mm, respectively. In the nasal angle area, the IT values were 0.459 ± 0.067 and 0.338 ± 0.051 mm, respectively. The mean IT values were 0.432 ± 0.060 and 0.340 ± 0.046 mm at 1 and 2 mm, respectively.

The association between IT and age, sex, and ocular morphological parameters (i.e., axial length, average corneal curvature, CCT, SS distance, and ACD) was assessed using linear regression analysis as shown in Table 2. At 1 mm, the parameters identified in the optimal models as predictors of IT were age (coefficient: 0.01) and average corneal curvature (0.12) at the temporal angle, and age (0.00), axial length (−0.02), and ACD (0.07) at the nasal angle. At 2 mm, the identified parameters were age (0.00) and average corneal curvature (0.07) at the temporal angle and ACD (0.00) at the nasal angle. The parameters identified in the optimal models to predict the average IT at the temporal and nasal angles were age (coefficient: 0.01), axial length (−0.02), average corneal curvature (0.01), and ACD (0.01) at 1 mm as well as age (0.00), average corneal curvature (0.09), ACD (0.06), and male (−0.02) at 2 mm.

**Table 2:** Parameters selected in the optimal models to explain the association between the IT and age, sex, and the ocular morphological parameters evaluated through a linear regression analysis based on the AICc index.

Selected predictor parameters						
	Age	Axial length	Average corneal curvature	ACD	Sex (male)	AICc
Dependent variables						
Temporal angle						
Iris thickness at 1 mm	0.01	-	0.12	-	-	−105.37
Iris thickness at 2 mm	0.00	-	0.07	-	-	−107.77
Nasal angle						
Iris thickness at 1 mm	0.00	−0.02	-	0.07	-	−107.96
Iris thickness at 2 mm	-	-	-	0.00	-	−125.18
Average of temporal and nasal angle						
Iris thickness at 1 mm	0.01	-0.02	0.01	0.01	-	−123.08
Iris thickness at 2 mm	0.00	-	0.09	0.06	−0.02	−137.73

Number in each cell represents the coefficient value.

**Fig 2:**
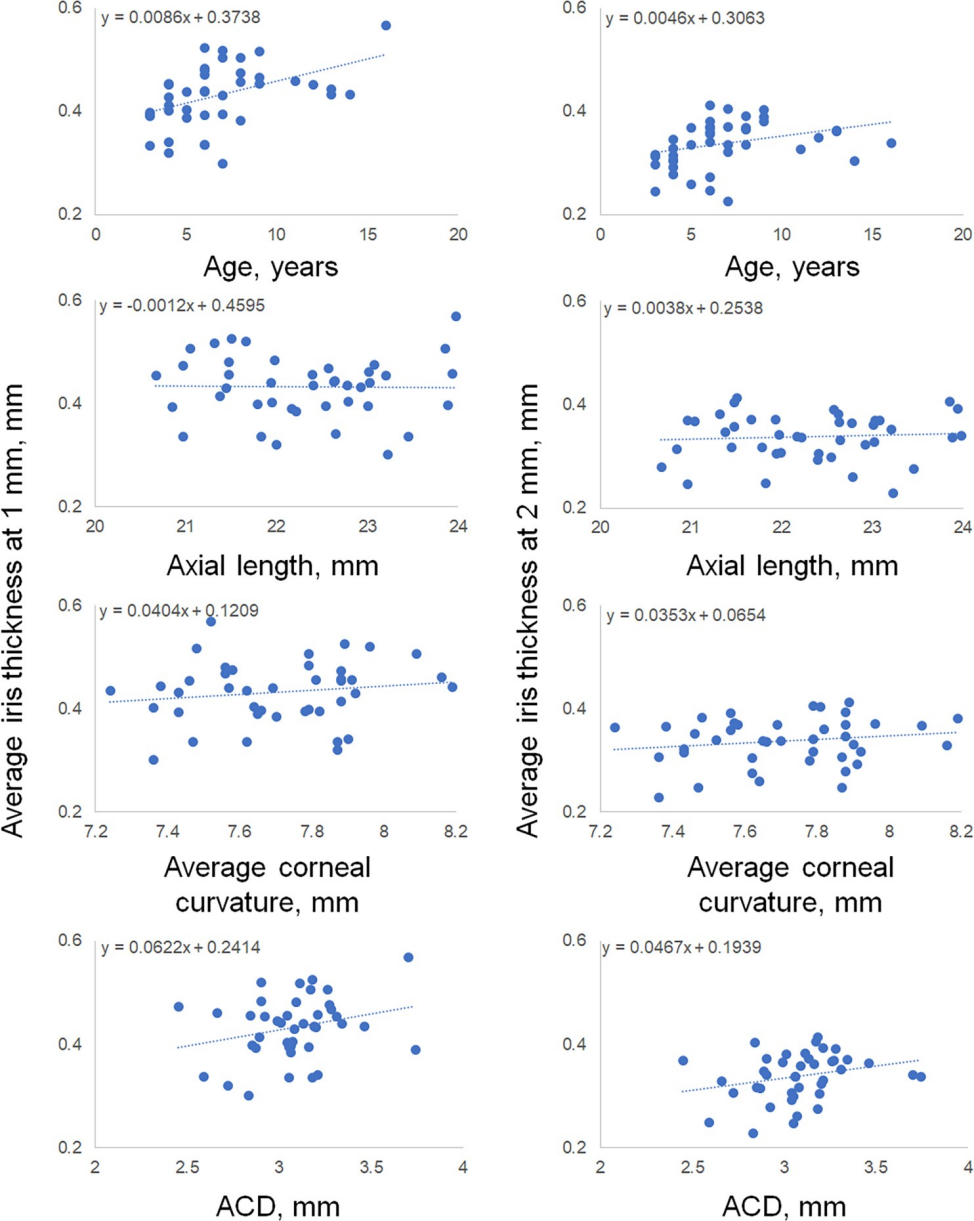
Association between the average IT and age, axial length, average corneal curvature, and ACD. Pearson’s correlation coefficient (r) between the average IT (temporal and nasal) and age was 0.45 and 0.31 at 1 and 2 mm, respectively (Fig 2 upper). The coefficient (r) between the average IT and axial length was −0.01 and 0.07, respectively (Fig 2 second line). The coefficient (r) between the average IT and average corneal curvature was 0.15 and 0.17, respectively (Fig 2 third line). The coefficient (r) between the average IT and ACD was 0.26 and 0.26, respectively (Fig 2 bottom).

The association between the average IT (i.e., temporal and nasal) and the identified parameters (i.e., age, axial length, average corneal curvature, ACD) are shown in Fig 2. Pearson’s correlation coefficient (r) between the average IT (temporal and nasal) and age was 0.45 (95% confidence interval [CI]: 0.16–0.66; P = 0.004) and 0.31 (95% CI: 0.01–0.56; P = 0.042) at 1 and 2 mm, respectively (Fig 2 upper). The coefficient (r) between the average IT and axial length was −0.01 (95% CI: −0.32–−0.29; P = 0.910) and 0.07 (95% CI: −0.23–−0.37; P = 0.643), respectively (Fig 2 second line). The coefficient (r) between the average IT and average corneal curvature was 0.15 (95% CI: −0.16–0.43; P = 0.341) and 0.17 (95% CI: −0.13–0.45; P = 0.273), respectively (Fig 2 third line). The coefficient (r) between the average IT and ACD was 0.26 (95% CI: −0.04–0.52; P = 0.096) and 0.26 (95% CI: −0.05–0.52; P = 0.099), respectively (Fig 2 bottom).

## Discussion

The current study showed the direct association between the IT in children and age. In addition, it implied an association with the ocular morphological parameters of axial length, average corneal curvature, and ACD. Thus, this study suggested the IT grows with age as well as other ocular structures. In this study, the IT was thicker in the nasal angle versus the temporal angle (Table 1). This tendency was previously demonstrated by Invernizzi A et al.[4] These investigators also showed that brown with some peripheral green eyes or brown eyes have thicker IT, whereas light blue eyes have thinner IT.[4] However, in our study, the participants belonged to a single race; therefore, the color of the iris did not affect the IT values. The development of the eyeball in children and the structural changes occurring in the uvea remain unknown. Twelker JD et al.[17] and Hashemi H et al.[18] reported that ACD, vitreous chamber depth, and axial length increased in parallel with the increasing age of children, especially between 6 and 9 years. In addition, corneal curvature did not change. However, the crystallin lens were thinned and flattened with increasing age.[17,18] Moreover, Jin P et al. reported that axial length, ACD, and all angle parameters increased with age in children.[12] Hashemi H et al. concluded that ocular development is mostly accomplished by the age of 14 years.[18] Regarding the CT in children, Jin P et al. showed that it was negatively associated with axial length and positively refractive error but not with age (7–13 years).[19] However, Xiong S et al. reported that the CT increased with age in children (3,001 Chinese children aged 6–19 years) with emmetropia and mild myopia (−0.5 D–−2.0 D). However, this increase was not observed in children with myopia (≤−2.0 D), and the axial length was negatively associated with CT in all refractive categories.[20] Read SA et al. reported that CT was positively associated with refractive error and age in children aged 10–15 years.[21] In summary, the axial length decreases CT, whereas age increases CT physiologically in children. However, rapid axial elongation and myopic shift counteract the effect exerted by age in children. Regarding the ciliary body, Pucker AD et al. showed that the ciliary body thickness (CBT) increases with age and myopia in children aged 6–14 years.[22] Furthermore, Bailey MD et al. reported that CBT increases with myopia and axial length in patients aged 8–15 years.[23]

The effect of axial length elongation on the CT and CBT in children is opposite, whereas that of age is similar. In our study, we showed that the IT increases with age (similar to the CT and CBT), average corneal curvature, and ACD. However, it decreases with axial length (similar to the CT). This is the first study to show a relationship between average corneal curvature, ACD and IT in children. Hashemi H et al. reported that, among the ocular morphological parameters, the highest correlation in children was observed between the axial length and corneal curvature (r = 0.699) as well as axial length and anterior chamber depth (r = 0.482).[18] This finding may support the present results obtained from the optimal regression model. Additionally, in the population study by Sng CC et al,[11] IT at 750 μm from scleral spur (which is near measurement point of IT at 2 mm in our study) was associated with male (−0.021), ACD (0.182), axial length (−0.032), and corneal curvature (0.017), which was consistent with our linear regression analysis.

The present study was characterized by limitations. First, this was a retrospective study with a small sample size. Further prospective studies involving large numbers of participants are warranted to confirm the various factors (i.e., ocular diseases, height, and body mass index) associated with IT in children.

Second, our method for the measurement of IT[5,6,7] (i.e., measured from the edge of the pupil) and that used also by Invernizzi A et al.,[10] (i.e., measured at the thickest point) were different from those employed in other previous studies[4,8,11] (i.e., measured near the angle). However, the root of the iris exhibits variations.[24] Notably, the iris stroma is loose connective tissue in free communication with the anterior chamber aqueous humor.[25] Therefore, the dynamic change due to the dilation of the pupil decreases the volume of the iris. Children have a larger pupil diameter than adults. Therefore, in children, measurement of the IT from the edge of the pupil may be a more reliable approach than that near the angle.

## Conclusions

IT in children increases with age. Additionally, IT was thinner with axial length and male and thicker in eyes with deeper anterior chamber and larger corneal curvature. Our study may partly explain the development of eyeball structures in children.

## Supporting information

S1 File All data. (XLSX)
